# Antibacterial Properties of Fucoidans from the Brown Algae *Fucus vesiculosus* L. of the Barents Sea

**DOI:** 10.3390/biology10010067

**Published:** 2021-01-19

**Authors:** Olga N. Ayrapetyan, Ekaterina D. Obluchinskaya, Elena V. Zhurishkina, Yury A. Skorik, Dmitry V. Lebedev, Anna A. Kulminskaya, Irina M. Lapina

**Affiliations:** 1 Petersburg Nuclear Physics Institute named by B.P. Konstantinov of National Research Center “Kurchatov Institute”, Mkr. Orlova Roshcha, 1, 188300 Gatchina, Russia; ayrapetyan_on@pnpi.nrcki.ru (O.N.A.); zhurishkina_ev@pnpi.nrcki.ru (E.V.Z.); lebedev_dv@pnpi.nrcki.ru (D.V.L.); kulminskaya_aa@pnpi.nrcki.ru (A.A.K.); 2 Kurchatov Genome Center—PNPI, Mkr. Orlova Roshcha, 1, 188300 Gatchina, Russia; 3 Faculty of Biotechnologies, ITMO University, Kronverksky Prospekt 49, Building. A, 197101 Saint Petersburg, Russia; 4 Federal State Budgetary Scientific Institution of Murmansk Marine Biological Institute of the Russian Academy of Sciences (MMBI RAS), Vladimirskaya, 17, 183010 Murmansk, Russia; obluchinskaya@mmbi.info; 5 Institute of Macromolecular Compounds of the Russian Academy of Sciences, Bolshoy Prospekt V.O. 31, 199004 Saint Petersburg, Russia; yury_skorik@mail.ru; 6 National Research Centre Kurchatov Institute, Akademika Kurchatova Square 1, 123182 Moscow, Russia

**Keywords:** *Fucus vesiculosus*, fucoidan, antibacterial activity, atomic force microscopy

## Abstract

**Simple Summary:**

The continuous emergence of new pathogenic strains of bacteria resistant to antibiotics highlights the importance of search for the new natural sources of antimicrobial compounds. Fucoidans, sulfated polysaccharides from the cell walls of brown algae, attract the attention of many researchers due to their wide range of biological activities. Being natural and practically non-toxic, they are considered as a promising antimicrobial component that could, where possible, replace the use of strong chemical antiseptics and modern antibiotics. Natural fucoidans are polydisperse polysaccharides whose composition can vary greatly depending on the species, habitat, as well as on the way of purification. In this work, we investigate the structure and antibacterial properties of fucoidans from the brown algae *Fucus vesiculosus* gathered in the littoral of the Barents Sea. These fucoidans appear to have a significant bacteriostatic effect on the growth of four tested bacterial strains. Such an effect was more pronounced for crude fucoidan samples that did not undergo additional steps of purification.

**Abstract:**

Fucoidans, sulfated polysaccharides found in cell walls of brown algae, are considered as a promising antimicrobial component for various applications in medicine and the food industry. In this study, we compare the antibacterial properties of two fractions of fucoidan from the brown algae *Fucus vesiculosus* gathered in the littoral of the Barents Sea and sampled at different stages of purification. The crude fraction of fucoidan was isolated from algae by extraction with aqueous ethanol and sonication. The purified fraction was obtained by additional treatment of the crude fraction with a solution of calcium chloride. The structural features of both fractions were characterized in detail and their antibacterial effects against several Gram-positive and Gram-negative bacteria were compared by photometry, acridine orange staining assay, and atomic force microscopy. Fucoidan inhibited growth in all of the above microorganisms, showing a bacteriostatic effect with minimum inhibitory concentrations (MIC) in the range between 4 and 6 mg/mL, with *E. coli* being the most sensitive to both fractions. Changes in the chemical composition after treatment of the crude fraction with a solution of calcium chloride led to a decrease in the content of sulfates and uronic acids and diminished antibacterial activity.

## 1. Introduction

Solving the problem of the continuous emergence of new pathogenic strains of bacteria resistant to antibiotics is one of the greatest challenges for modern medicine. Currently antibiotic-resistant infections cause about 700,000 deaths each year and, according to some forecasts, the global mortality rate from resistant bacteria could rise up to 10 million per year [1,2]. To reverse this trend requires not only design of new chemical antimicrobial substances, but also an intensive search for new natural antimicrobial agents that could alleviate the current dependence of the modern medicine on traditional antibiotics. Brown algae, the cell walls of which contain sulfated polysaccharides, fucoidans, are being intensively studied as a natural source of biologically active substances. The unique property of these biopolymers is the presence of a wide range of biological activities: antiviral, antibacterial, anticoagulant, immuno-stimulating, anti-inflammatory, and antitumor [3,4].

Various preparations containing fucoidans as a biologically active component are being developed for medical use, for example, in wound dressings [5,6]. A lack of toxicity together with bacteriostatic properties also allowed their application in the food industry, increasing the shelf life of products and not killing the natural human microflora when ingested. For example, the use of ultrasonic micronization of fucoidan as a food ingredient for the preparation of lactic acid products has been recently reported [7].

One of the extensively studied fucoidan producent is *Fucus vesiculosus* Linnaeus (Phaeophyceae, Fucales), a widespread specimen of brown algae that grow in cold and moderately cold waters along the rocky shores in the northern hemisphere [8,9]. Due to the eurytopic nature of this alga, the phytochemical composition of *F. vesiculosus* varies significantly depending on its ontogeny and habitat [10,11]. A distinctive feature of *F. vesiculosus* from the Barents Sea is a high content of fucoidan (from 10% to 14%, and up to 18% in desalinated bays) together with a low content of laminaran (no more than 2–3%) [12,13]. The content of polyphenols in *F. vesiculosus* on the eastern coast of the Barents Sea ranges from 12% to 18%, which is slightly higher than that of the same species in the neighboring Norwegian Sea (9–11%) [14,15,16].

Natural fucoidans are highly polydisperse polysaccharides so that their chemical and biological properties can be affected by purification procedure [3,11]. Moreover, since fucoidans are isolated from seaweed by water or alcohol extraction, they usually contain impurities of other substances that may contribute to the biological activity. For example, the antioxidant activity of fucoidans is associated with the polyphenolic impurities, which form stable complexes with sulfated polysaccharides that cannot be destroyed without disrupting the integrity of fucoidan molecules [17]. In this work, we continued our recent studies of the biological activity of fucoidans from *F. vesiculosus* algae collected in the littoral of the Barents Sea [12,16], and investigated the antibacterial properties of these compounds sampled at two consecutive stages of purification.

## 2. Materials and Methods

All reagents were purchased from Sigma-Aldrich (Germany) and Acros Organics (USA), unless otherwise specified.

Preparation of fucoidans: The thalli of *F. vesiculosus* L. collected in the autumn hydrological period (September 2018) in the Zelenetskaya Bay of the Barents Sea were used as raw material. The algae were frozen and freeze-dried. Shredded algae were sequentially treated with an organic solvent, ethyl alcohol, alcohol-water mixtures in accordance with a patented technological scheme [18,19,20]. The prepared algae were extracted by percolation with a 5% ethanol solution at pH 4 with preliminary ultrasonic treatment. The ultrasonic device “Volna” (OOO “Center of Ultrasonic Technologies”, Biysk, Russia) was used as a source of ultrasound. The extract was concentrated on an AR-3–100 PS ultrafiltration equipment (NTP Biotest, Kirishi, Russia), and lyophilized, as a result, crude fucoidan F with a yield of 10 ± 2% was obtained. A purified sample of sulfated polysaccharides Fo was obtained in the following way: 1 g of crude fucoidan F was dissolved with stirring in 60 mL of 2% CaCl_2_ solution and dialyzed with a 3.5 kDa pore dialysis membrane (Spektra/Por, Canada) against distilled water for 12 h [3,21]. The insoluble part was removed by centrifugation at 8000 rpm for 1 h, the supernatant solution was lyophilized. The yield of purification was 64.5 ± 2.5% of the crude fucoidan by weight.

The monosaccharide composition of fucoidans was evaluated by HPLC method [22]. Briefly, the samples were hydrolyzed with a 2M trifluoroacetic acid for 6 h and 100 °C. The hydrolyzed polysaccharides were cooled in ice water bath and centrifuged at 2300× *g* (3500 rpm) for 15 min at 25 °C (Elmi CM-6M, Elmi Ltd., Riga, Latvia). The supernatant fraction was neutralized to pH 7 with 2 M NaOH. The obtained samples were analyzed on LC-10A chromatograph equipped with an RID-10A detector (Shimadzu Corp., Kyoto, Japan). Samples were injected into a Shodex Asahipak NH2P-50 4E 4.6 × 250 mm column (Showa Denko Co., Tokyo, Japan) at 50 °C and eluted with 0.25 M orthophosphoric acid and acetonitrile in a 20:80 ratio (1.0 mL/min).

The protein content was determined by the Bradford method [23]. Bovine serum albumin was used as a standard.

The sulfate content was determined by the turbidimetric method after acidic hydrolysis of the samples with a 4 N HCl solution at 100 °C for 6 h using K_2_SO_4_ as a standard [24].

The amount of fucose was determined by a modified Dische method [25] with L-cysteine (Merck, cat. no K32296038 Lot. 330) and sulfuric acid (Neva-reactiv, 98% purity), using L-fucose (Fluka, cat. No 47870) as a reference [26].

The content of phenolic compounds was determined as described in [27] with some modifications. The fucoidan samples were prepared with concentration 10 mg/mL in distilled water. A 10 µL aliquot of sample was added to 50 µL of Folin–Ciocalteu reagent and 200 µL of 20% sodium carbonate and the mixture was then diluted with 90 µL of distilled water. After incubation for 10 min at 70 °C, samples were put on ice to stop the reaction. Absorbance was read at 700 nm on a JASCO V 560 spectrophotometer (JASCO Corporation, Tokyo, Japan). Phloroglucinol was used as the standard. Contents of phenolic compounds were expressed as a percentage (in dry weight basis) of the total mass.

Nuclear magnetic resonance (NMR) spectra were obtained on an Avance DPX-300 NMR spectrometer (Bruker, Billerica, MA, USA) at 50 °C. The sample concentration was 20 mg of polysaccharide per ml of D_2_O.

FTIR spectra were obtained using a Vertex-70 spectrometer (Bruker, Bremen, Germany) equipped with a Pike attenuated total reflectance accessory (a ZnSe prism). The Bruker OPUS spectroscopy software was used for data acquisition and analysis.

The molecular weight distribution of fucoidans was determined using HPLC [28]. An LC-20A chromatograph with a RID-10A refractive index detector (Shimadzu Corp., Kyoto, Japan) with a Shodex Asahipak GS-520 HQ and GS-620 HQ (7.5 mm × 300 mm) was used. Fucoidan samples were dissolved in highly purified water and filtered through a membrane filter (0.45 μm pore size). Polysaccharides were separated over successively connected columns of Shodex Asahipak at 60 °C with elution by H2O (0.5 mL/min). Columns were calibrated using standard pullulans of MWs from 6.2 to 740 kDa (P-82 kit, Showa-Denko Co., Tokyo, Japan) and blue dextran-2000 (Sigma-Aldrich). The molecular mass distribution of fucoidan was assessed by normalizing the peak areas.

The dynamic viscosity was determined using a Brookfield DV2T viscometer, measuring cone CPA—41Z (Brookfield Engineering Labs., Inc., Middleborough, MA, USA). Solutions of fucoidans F and Fo with concentrations of 5, 10, and 20 mg/mL were analyzed at 22 °C.

Antibacterial activity of fucoidans was tested against *Escherichia coli* strain BL 21, *Staphylococcus epidermidis* 4a (in-house laboratory collection), *Staphylococcus aureus* strain AR 3916, and *Bacillus licheniformis* strain DSMZ 8782. These strains were stored in cryogenic capsules at −70 °C and for routine works were maintained in Nutrient Broth (NB) at 37 °C in an aerobic atmosphere for 24 h. To determine minimum inhibitory concentrations (MIC), inhibition percentage and growth curves, the microorganisms were grown overnight on NB medium at 37 °C. Fucoidan samples were dissolved in medium to the final concentrations of 2, 4, 6, 8 and 10 mg/mL. The concentration of bacterial cells was 1 × 10^5^ CFU/mL. An aliquot of each bacterial suspension (10 μL) was added to the wells of a 96-well plate containing 150 μL of the medium. Following the incubation for 24 h at 37 °C, the sample turbidity in the serially diluted cultures was monitored. The MIC was determined as the lowest concentration of fucoidan that completely inhibited a visible growth of the tested microorganisms according to the broth microdilution method guided by the Clinical and Laboratory Standards Institute (CLSI) [29].

Growth inhibition curves were registered by photometric assay of red light absorbance using the same set of fucoidan concentrations. Preliminary tests included 8 additional strains (*B. subtilus* BP 168, *B. cereus* BP 170, *Klebsiella pneumonica sub.sp* ATCC 13883, *Enterococcus avia* B7786; *B. cereus* SP, *B*. *subtilus* K51, *B*. *cereus* 4b, and *Pasteurella multocida* from in-house laboratory collection) and were performed using 16 and 24 mg/mL fucoidan concentrations. The percentage of bacterial growth inhibition was calculated using the formula:

Growth inhibition = (1 − (Ac/Ao)) × 100, where Ac is the optical density of the sample at the concentration of fucoidan (c) and Ao is the optical density of the negative control.

To obtain the growth curves, the samples were incubated at 37 °C with shaking at 200 rpm for 24 h, while the number of viable cells was determined every 2 h by photometry.

The absorbance was measured at 620 nm using a Microplate reader Multiscan FC Thermo Fisher Scientific type 357 (China). Gentamicin solution (10 μg/mL) was used as positive control, and the negative control used for the study was prepared without fucoidan.

Staining with acridine orange was performed according to the method described in [30] with slight modifications. The microorganisms were grown overnight on LB medium at 37 °C. Fucoidan samples were dissolved in medium to give final concentrations, corresponding to the MIC. Bacteria were seeded in a 24 well plate with a final concentration of 1 × 10^5^ CFU/mL. After 24 h of incubation, the cells were harvested and centrifuged at 5000 rpm for 5 min. The resulting bacterial suspension was mixed with 0.5 mL of 0.01% acridine orange (AO) in McIlvaine buffer. After 15 min incubation at room temperature, the samples were centrifuged (5000 rpm for 5 min), the resulting pellets resuspended in 0.5 mL PBS to remove excess dye, and centrifuged again. The pellets were resuspended in 0.4 mL of PBS, mixed, and an aliquot was applied onto a glass slide and analyzed on EVOS FLoid microscope (Life Technologies, Carlsbad, CA, USA).

The test with resazurin was carried out according to the method [31] with slight modifications. Briefly, the microorganisms were grown overnight on NB medium at 37 °C. Fucoidan samples were dissolved in medium to give final concentrations corresponding to the MIC. The concentration of bacterial cells was 1 × 10^5^ CFU/mL. An aliquot of each bacterial suspension (10 μL) was added to the wells of a 96-well plate containing 150 μL of medium. These cultures were then incubated at 37 °C for 24 h. Then 10 μL of resazurin with a concentration of 16.8 mg/mL was added to each well. The plate was incubated for 4 h at 37 °C. The colour change was then assessed visually. Any shades of red were recorded as positive.

AFM measurements of cell integrity and surface topology were performed according to the method described in [32]. The microorganisms were grown overnight on NB medium at 37 °C. Fucoidan samples were dissolved in medium to give MIC concentrations. The concentration of bacterial cells was 1 × 10^5^ CFU/mL. An aliquot of each bacterial suspension (10 μL) was added to the wells of a 24-well plate containing 1 mL of medium. These cultures were then incubated at 37 °C for 24 h. After incubation 0.5 mL of culture broth was centrifuged for 5 min at 13,000 rpm. The bacterial suspension was then washed in 0.9% NaCl solution. Ten μL of sample was applied to the clean glass slide, dried in air, gently washed twice using 2.5 mL of distilled water, and air-dried again. The scanning was performed in a semi-contact mode on the SolverNext atomic force microscope (NT-MDT, Zelenograd, Russia) under the NovaPx program (production of NT-MDT) using an NSG01/Co cantilever probe. The resulting images were processed in the Gwyddion software.

Statistical processing: For statistical data processing, plotting charts, and diagrams, Excel 2010 (Microsoft) and OriginPro 16 (Microcalc) were used. The graphs and tables show average values of at least 3 independent experiments, bars represent the standard errors. The statistical significance of the observed differences was assessed using Student’s *t*-test.

## 3. Results

### 3.1. Characterization of Fucoidans

Crude fucoidan F was isolated from *F. vesiculosus* brown algae of the Barents Sea by extraction with 5% ethanol solution at pH 4 and ultrasonic treatment according to a previously described patented method [20]. The obtained polysaccharide had a high content of fucose, uronic acids and polyphenols. The purified sample Fo was prepared by treatment of fucoidan F with a 2% CaCl_2_ solution and contained almost 10% more fucose (by weight) than the crude F according to spectrophotometric data [25]. However, according to the molar ratio of monosaccharides obtained by HPLC, the amounts of such major monosaccharides as fucose, mannose, and glucose decreased, while xylose and galactose increased. The content of sulfates (from 27.6% in F to 18.3% in Fo) and uronic acids (from 9.2% in F to 5.1% in Fo) was also significantly decreased in the purified sample. The results of the analysis of the composition of both samples are presented in Table 1.

The groups of signals corresponding to the anomeric H1 protons (5.1–5.5 ppm), as well as those characteristic of the protons of the H2–H5 carbohydrate ring (3.3–4.9 ppm) were clearly distinguishable in the 1H NMR spectra (Supplementary Figure S1) of both fractions. The presence of α-L-fucose residues was confirmed by intense signals in the high-field region (1.1–1.4 ppm), typical of the H6 α-L-fucopyranosides. Peaks at 2.2–2.3 ppm indicate the presence of acetyl groups in the structure of both fucoidans.

**Figure 1:**
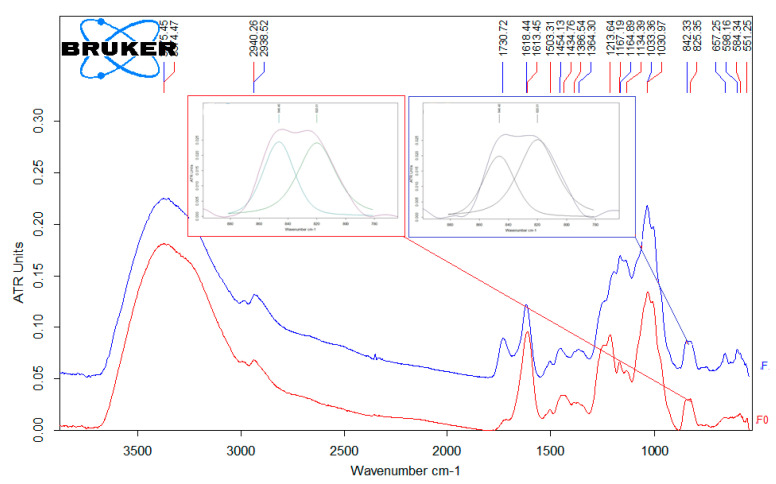
IR spectra of the crude fucoidan (F) and purified fucoidan (Fo).

IR spectra of both crude (F) and purified (Fo) fucoidan samples had intensive absorption bands corresponding to S=O bond at 1164 and 1213 cm^−1^, respectively (Figure 1). The peak at 1730 cm^−1^ could be related to the higher amount of uronic acids in the chemical structure of the crude sample. When analyzing the peak bands at 842 cm^−1^ for F and 825 cm^−1^ for Fo, we found that they can be described by two components with maxima at 846 and 820 cm^−1^. In the case of the sample F, the ratio of S846/S820 was 0.77, and in the IR spectrum of Fo the S846/S820 ratio was 0.6, indicating the prevailing localization of the sulfo-groups in the equatorial position of both polysaccharides.

The results of the molecular mass distribution measurements for fucoidans F and Fo are presented in Table 2.

Both samples exhibited high polydispersity in the molecular weights. Chromatogram analysis of fucoidan F revealed 5 peaks ranging from more than 2.5 MDa to 10 kDa. The chromatograms of the purified Fo sample showed four peaks which positions were shifted toward the lower molecular weights. The average Mw for F and Fo was 735 kDa and 343.8 kDa, respectively.

Comparative analysis of dynamic viscosity of fucoidans F and Fo at the same polysaccharide concentrations (Supplementary Figure S2) revealed that values for the viscosities of Fo were significantly lower than for F (*p* < 0.01), which was in agreement with the chromatographic data showing lower molecular weight of Fo.

To summarize the results of the experiments, fucoidans F and Fo can be characterized as acetylated α-L-fucans highly heterogeneous in molecular weight and containing similar amounts of polyphenols, but differing significantly in the content of sulfates, monosaccharides, and uronic acids.

### 3.2. Antibacterial Assays

A preliminary photometric test of the antibacterial activity of fucoidans F and Fo performed on 12 different bacterial strains has shown a near complete (>90%) inhibition of growth by the presence of either of the fucoidan fractions in the medium at 16–24 mg/mL concentrations (Supplementary Figure S3). A single exception was the moderate (ca. 60%) inhibition of growth of the pathogenic *Klebsiella pneumonica* by the purified fucoidan fraction. For a more detailed investigation one Gram-negative (*E. coli*) and three Gram-positive (*B. licheniformis*, *S. aureus*, and *S. epidermidis*) bacterial strains were selected to cover different cellular morphologies and envelope organizations.

Both of the sulfated polysaccharide fractions showed an inhibitory effect on the growth of each of the four bacteria tested with MIC ranging from 4 to 6 mg/mL (Table 3). *E. coli* exhibited the highest sensitivity to both of the fucoidan samples, while *B. licheniformis* was the least sensitive. Minimum bactericidal concentration (MBC) values were not determined since no bactericidal effect was observed at a polysaccharide concentration below 24 mg/mL, while at higher concentrations the fucoidans were poorly dissolved in the medium.

**Figure 2:**
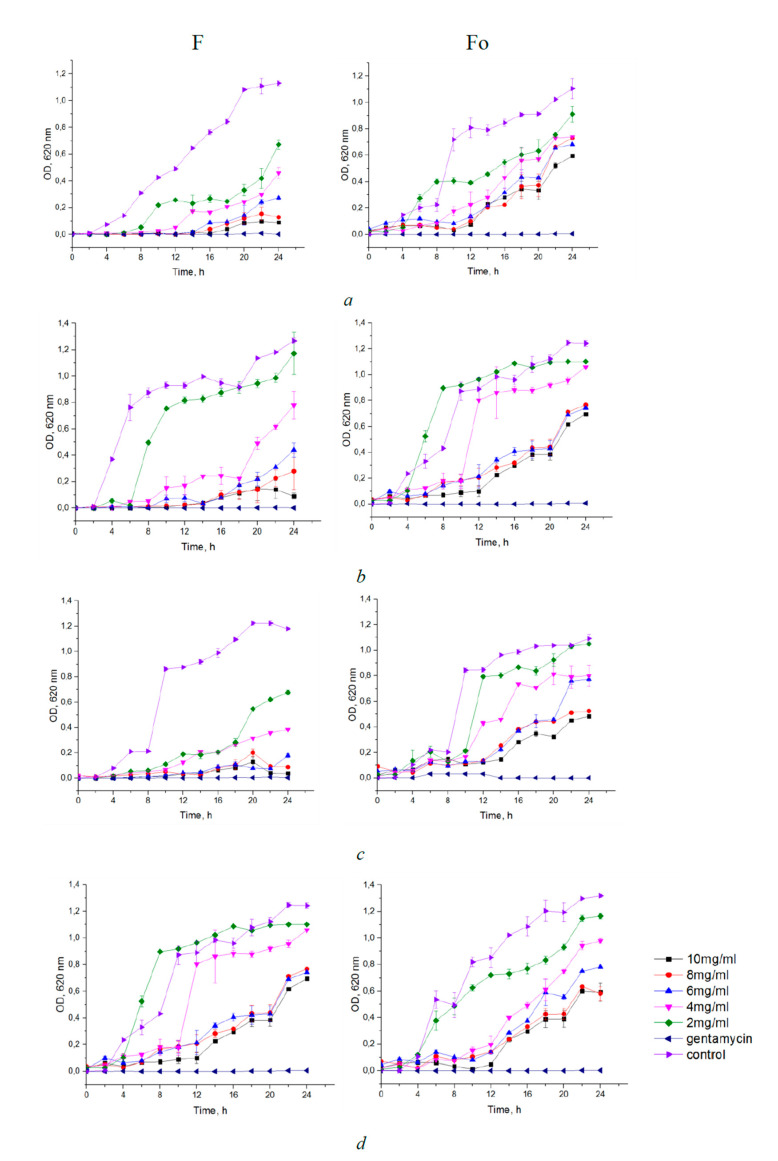
Growth curves of bacteria treated with fucoidans F and Fo for 24 h ((**a**)—*E*. *coli*, (**b**)—*B*. *licheniformis*, (**c**)—*S*. *aureus*, (**d**)—*S*. *epidermidis*). Each value is the mean ± SD of three replicates.

**Figure 3:**
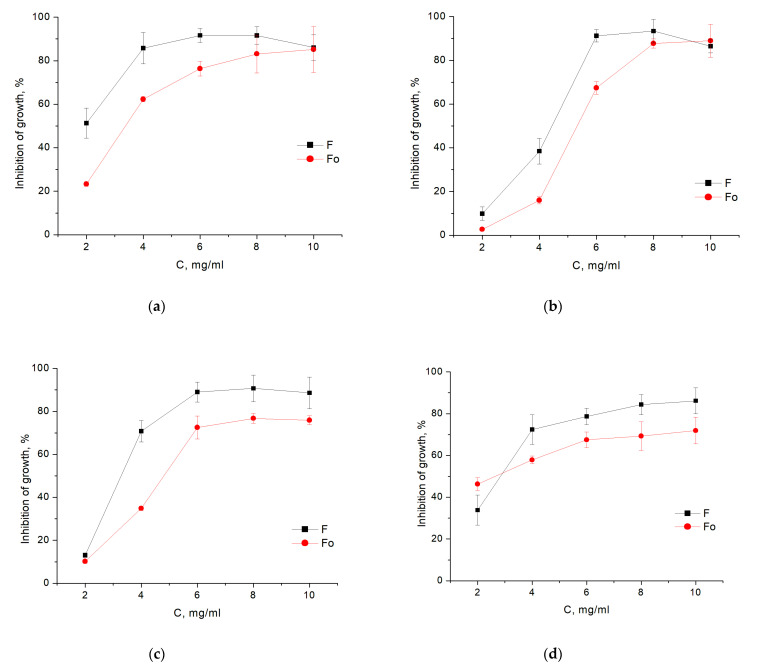
The percentage of growth inhibition of bacteria treated with different concentrations of fucoidans F and Fo after incubation for 24 h ((**a**)—*E*. *coli*, (**b**)—*B*. *licheniformis*, (**c**)—*S*. *aureus*, (**d**)—*S*. *epidermidis*). Showed the percentage of inhibition of growth of bacteria in fucoidan-containing medium with respect to bacteria grown without fucoidans. Each value is the mean ± SD of three replicates.

The growth inhibition of bacteria incubated with different concentrations of fucoidans F and Fo in the range of 2–10 mg/mL for 24 h was also determined. Analysis of the growth curves of bacteria taken for 24 h showed a dose-dependent effect of F or Fo fucoidans on bacterial strains (Figure 2). In general, crude fucoidan F exhibited a greater inhibitory effect compared to purified Fo. *E. coli* and *S. aureus* were most sensitive to crude fucoidan F, and their growth was noticeably inhibited by fucoidan F at concentrations as low as 2 mg/mL. The inhibitory effect of purified fucoidan Fo on the growth of the same bacteria was significant only at concentrations 4 mg/mL and above. Figure 3 shows the percentage of inhibition of growth of bacteria in fucoidan-containing medium with respect to bacteria grown without fucoidans. It should be noted that the MIC of fucoidans corresponds to the inhibition of bacterial growth by more than 60% relative to the control.

**Figure 4:**
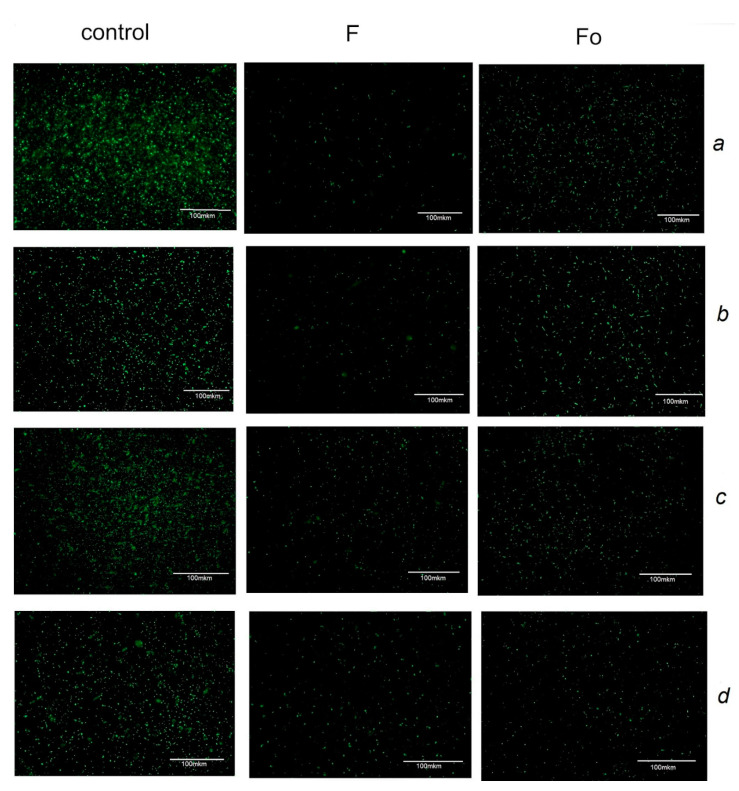
Fluorescent microscopy analysis of bacteria treated by fucoidans F and Fo for 24 h ((**a**)—*E*. *coli*, (**b**)—*B*. *licheniformis*, (**c**)—*S*. *aureus*, (**d**)—*S*. *epidermidis*).

Fluorescence microscopy assay using acridine orange, a nucleic acid binding dye that binds selectively to living cells and emits green fluorescence, also showed antimicrobial effect of fucoidans F and Fo. In all four bacterial cultures treated with MIC of fucoidans F and Fo, we observed a decrease in the number of acridine orange stained bacteria (Figure 4). The treatment by fucoidan F resulted in the smaller number of live cells determined by acridine orange assay as compared to fucoidan Fo for all bacterial species except *S. epidermidis*.

**Figure 5:**
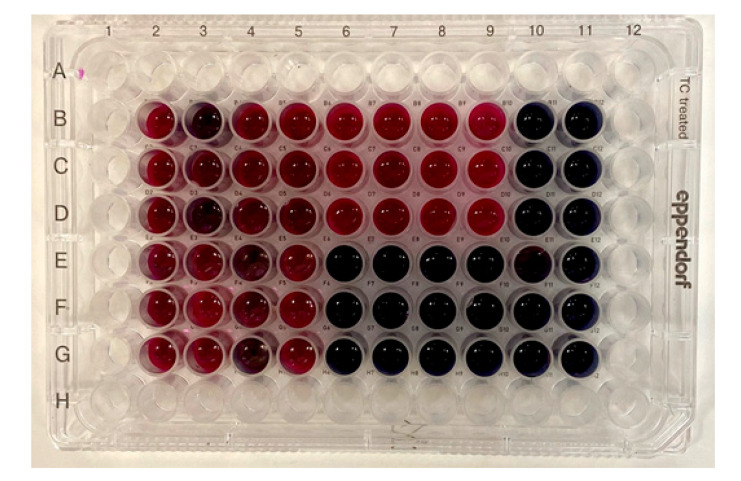
Plates after 24 h incubation with fucoidans F and Fo in resazurin assay. Red colour indicates growth and blue means inhibition of growth. Test (fucoidan in MIC + bacteria + NB + indicator): (B2–D2: F + *E.coli*; B3–D3: F + *B. licheniformis*; B4–D4: F + *S. aureus*; B5–D5: F + *S. epidermidis*; E2–G2: Fo+ *E.coli*; E3–G3: Fo + *B. licheniformis*; E4–G4: Fo + *S. aureus*; E5–G5: Fo + *S. epidermidis*). Sterility control (fucoidan + NB + indicator), no bacteria: (B10–D10: F—4 mg/mL; B11-D11: F—6 mg/mL; E10–G10: Fo—4 mg/mL; E11–G11: Fo—6mg/mL). Positive control (bacteria + gentamicin + NB + indicator): (E6–G6: *E.coli*; E7–G7: *B. licheniformis*; E8–G8: *S. aureus*; E9–G9: *S. epidermidis*). Negative control (bacteria + NB + indicator): (B6–D6: *E.coli*; B7–D7: *B. licheniformis*; B8–D8: *S. aureus*; B9–D9: *S. epidermidis*).

After the growth of bacteria was stopped by 24 h incubation in the media containing sulfated polysaccharides at MIC, it could be resumed by subculturing them on agar fucoidan-free media (data not shown), indicating that fucoidans F and Fo exhibit mainly bacteriostatic effect against the studied bacteria. To further investigate the effect of fucoidans F and Fo on the viability of the tested microorganisms, a test with resazurin, a redox indicator, used to assess cell growth in various cytotoxicity assays [31], was performed. It was shown that bacteria retained the function of mitochondrial respiration after treatment with fucoidans F and Fo in doses of MIC for 24 h (Figure 5), which confirmed our assumption that the investigated fucoidans F and Fo showed bacteriostatic rather than bactericidal activity.

**Figure 6:**
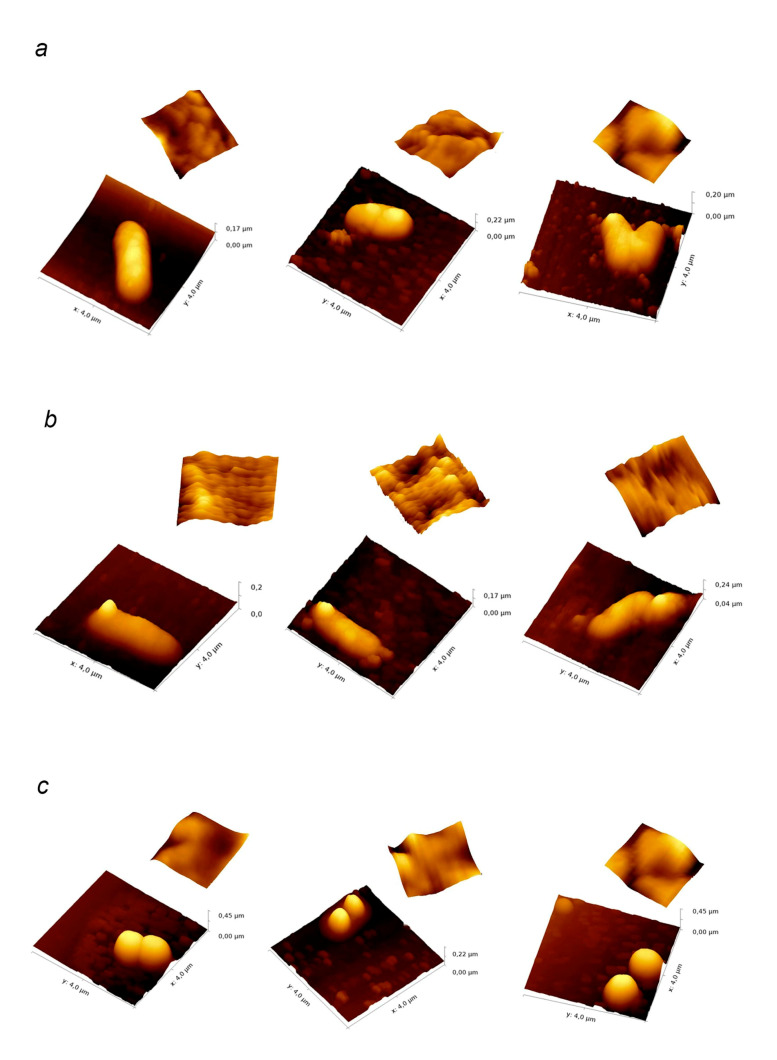

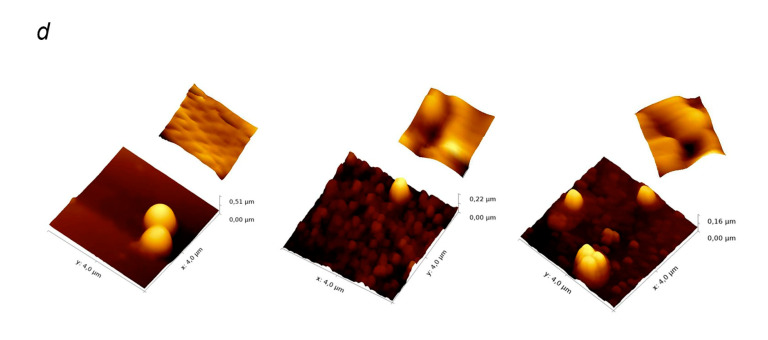
(**a**,**b**). AFM images of bacteria treated with fucoidan F and Fo for 24 h ((**a**)—*E*. *coli*, (**b**)—*B*. *licheniformis*): (**left**) control; (**middle**) treated with fucoidan F; (**right**) treated with fucoidan Fo. Inserts show the surface topograms in 0.5 × 0.5 μm field. (**c**,**d**). AFM images of bacteria treated with fucoidan F and Fo for 24 h ((**c**)—*S*. *aureus*, (**d**)—*S*. *epidermidis*): (**left**) control; (**middle**) treated with fucoidan F; (**right**) treated with fucoidan Fo. Inserts show the surface topograms in 0.5 × 0.5 μm field.

We analysed morphological changes in bacteria after their treatment with fucoidans F and Fo at MIC for 24 h using atomic force microscopy. All tested bacteria retained their integrity (Figure 6a–d). We observed a significant decrease in the size of *S. aureus* and *S. epidermidis* when subjected to fucoidan treatment (Figure 6c,d). For *S. epidermidis* the height of the bacterial cell decreased from 0.47 ± 0.04 μm to 0.23 ± 0.01 μm and 0.19 ± 0.03 μm and the cell diameter decreased from 0.91 ± 0.08 µm to 0.63 ± 0.06 µm and 0.47 ± 0.08 µm after treatment with F and Fo fucoidans, respectively. Similar effect was observed for *S. aureus*, where treatment with fucoidans F and Fo resulted in the diameter decrease from 1.0 ± 0.1 µm to 0.63 ± 0.08 µm (F) and 0.61 ± 0.08 μm (Fo) and the height decrease from 0.50 ± 0.03 μm to 0.29 ± 0.07 μm (F) and 0.45 ± 0.04 μm (Fo). At the same time, no significant change in size of rod-shaped microorganisms (Figure 6a,b). While the average height of *B. licheniformis* cells treated with crude fucoidan F appeared to decrease slightly from 0.19 ± 0.03 to 0.17 ± 0.01 µm, neither of fucoidans had any significant effect on the size of *E. coli* cells (the average height of the untreated bacteria—0.18 ± 0.03 μm, after treatment with F—0.18 ± 0.03 μm; after treatment with Fo—0.19 ± 0.05 μm). We have also observed a significant increase in the surface roughness parameter for the gram-positive bacteria after fucoidan treatment (Table 4). Both fucoidan samples had a similar effect on this parameter, except in *S. epidermidis* where no effect of Fo was observed. Similar to the cell size, the surface roughness of *E. coli* was not affected by either of the fucoidan samples.

## 4. Discussion

The main structural characteristics of fucoidan, such as molecular weight, monomer composition, content of sulfate groups, and their position, can change under the influence of extraction processes, as well as seasonal and geographical factors [11,33]. When studying seasonal fluctuations in the chemical composition of fucoidan from *F. vesiculosus* of the coast of Aberystwyth Great Britain, it was found that the average fucose content varied from 26 to 39 wt%, and the sulfate content from 9 to 35 wt% during the year [34]. Our earlier studies have shown that while the highest fucoidan content in *F. vesiculosus* of the coast of Barents Sea was observed in the autumn hydrological period (August—October) [35], the content of polyphenolic compounds in this period was lower than during summer [14,36].

Fucoidan purification methods can also affect its properties. Therefore, when studying the biological activity of sulfated polysaccharides, every particular sample of fucoidan has to be characterized in detail, so that the relationship between its chemical and biological properties could be identified.

In our study, the chemical composition of crude fucoidan F and its purified fraction Fo obtained by treatment with a CaCl_2_ solution, had substantial differences. Due to precipitation of calcium salts of alginates during purification procedure, the content of uronic acids decreased by almost two times, from 9.2% in fucoidan F to 5.1% in Fo (Table 1). Removal of a part of alginates from the composition affected both the molecular weight distribution and the viscosity of the polysaccharide. The average molecular weight and viscosity of the purified fucoidan have significantly decreased as compared to the untreated fucoidan (Table 2, Supplementary Figure S2). Apparently, alginates made up a significant part of the removed high molecular weight fraction.

Treatments with CaCl_2_ also resulted in selection of the fucoidan fractions with higher xylose and galactose and lower fucose and mannose content. However, removal of the alginates and other impurities resulted in the overall increase in the mass fraction of fucose, similar to that observed in other studies [3,16,33]. Treatment of crude fucoidan F with a solution of calcium chloride did not lead to a noticeable change in the amount of polyphenols in the purified sample (6.8 ± 0.4% and 6.5 ± 0.6% for F and Fo, respectively), since polyphenols do not precipitate in aqueous salt solutions. Complete separation of sulfated polysaccharides from polyphenolic impurities is a difficult task, as it was shown, for example, in [37], where hydrogen peroxide was used to isolate fucoidans from a natural complex with polyphenols. The sulfate content of the purified fucoidan significantly decreased (from 27.6 ± 0.2% in F to 18.3 ± 0.3% in Fo), while according to IR spectroscopy data the position of sulfate groups at C2 and C4 of the fucose residue (predominantly equatorial) did not change. Since fucoidan is a polydisperse mixture of sulfated polysaccharide molecules that differ in size and chemical composition [38], it seems that the change in the ratio of monosaccharide content in the purified sample is caused, among other things, by the loss of some polysaccharide fractions because of dialysis.

The degree of sulfation can be one of the main parameters that influences the antimicrobial activity of polysaccharides. For example, in a study [39], among the fucoidans isolated from five different samples of brown algae (*Hizikia fusiforme*, *Kjellmaniella crassifolia*, *Laminaria japonica*, *Sargassum honeri* and *Undaria pinnatifida*) only sulfated polysaccharides isolated from *U. pinnatifida* and *K. crassifolia* exhibited inhibitory activity against the tested bacteria at a maximum test concentration of 1000 µg/mL. The sulfate content in all five obtained polysaccharides was relatively low (from 5.2% to 8.6%), since the authors may have chosen a rather hard extraction method (heating at acidic pH), which led to the loss of sulfates, as a consequence, to a decrease in the antimicrobial activity of fucoidans. In the same study, of the two samples of commercial fucoidan from *F. vesiculosus* (Sigma-Aldrich Inc., St. Louis, MO, USA), in which the sulfate content differed by almost two times (14% versus 7.5%), only fucoidan with a higher degree of sulfation showed a noticeable antimicrobial activity against a number of Gram-positive bacteria.

The role of uronic acid levels in the composition of fucoidans in their antibacterial activity is not so obvious. The study of fucoidan isolated from *L. japonica* [40], depolymerized by autoclaving and separated into fractions with different molecular weights, have showed that the low molecular weight fraction (<6 kDa), having the highest content of uronic acids (28.4%) compared to the initial fucoidan and the other fractions (from 15.9% to 18.1%), exhibited the highest antibacterial activity. At the same time, the content of sulfates in this fraction (38.4%) was slightly higher than that in the remaining samples (from 33.7% to 36.2%). On the other hand, Ashayerizadeh et al. [41] reported the effect of using two methods of aqueous extraction of fucoidan from Sargassum tenerrimum on its chemical composition and properties and showed a greater antibacterial activity in the sample with lower uronic acid content (13.15% versus 16.3%) while the content of sulfates and the molecular weight distribution of both fractions being similar. In our work, crude fucoidan F, having contents of sulfates and uronic acids 9.3% and 4.1% higher than those in the purified Fo samples, showed a greater degree of antibacterial activity.

The growth of all of the studied microorganisms was inhibited by the presence of both F or Fo fucoidans in the cultivation medium (Figure 2, Figure 3 and Figure 4). The MIC values (Table 3) for *E. coli* (F and Fo: 4 mg/mL) and *S. aureus* (F: 4 mg/mL; Fo: 6 mg/mL) were lower than those reported in [40] for depolymerized fucoidans from *L. japonica* (*E. coli*: MIC 6.25 mg/mL, MBC 10.0 mg/mL; *S. aureus*: MIC 8.0 mg/mL, MBC 12.5 mg/mL), but higher than for the purified fucoidan fraction from *Sargassum polycystum* (*E. coli*: MIC 200 µg/mL, MBC 300 µg/mL; *S. aureus*: MIC 200 µg/mL, MBC 300 µg/mL) [30]. Our results indicate that additional purification step commonly used in fucoidan processing does not appear to yield any enhancement in terms of antibacterial activity. The observed bacteriostatic effect of the purified fraction was lower than that of the crude fucoidan. There appeared to be no gain in the selectivity toward potentially pathogenic species. This could be explained either by significant changes in the chemical composition introduced during purification, particularly the decrease in sulfates and uronic acids, or by the loss of the impurities contributing to the bacteriostatic action.

Growth inhibition was confirmed by growth curves and acridine orange staining assay (Figure 2 and Figure 4). Bacteria resumed their growth when subcultured on fucoidan-free agar medium that points to bacteriostatic rather than bactericidal effect of fucoidans. The bacteriostatic effect of fucoidans was confirmed by a test with the redox indicator resazurin, which showed that the studied bacteria retain their viability after their treatment with fucoidans [31].

Atomic force microscopy can be a powerful tool for a detailed elucidation of the action of antibacterial compounds that allows determining their effects on the surface integrity, surface roughness and mechanical stiffness [42]. For example, in [43] the antimicrobial activity of chitosan derivatives with different molecular weights was studied using AFM in two microorganisms, *E. coli* and *S. aureus*. The images obtained made it possible to reveal not only antibacterial effects, such as mechanical changes in the bacterial cell wall caused by treatment, but also response strategies used by the bacteria: cell wall collapse in case of cell death or clustering of surviving bacteria. To this day, there are few, if any, works where AFM was used to study morphological changes in bacteria treated with fucoidans.

An assessment of the morphological changes in bacteria exposed to fucoidans F and Fo using atomic force microscopy has revealed that the integrity of all bacterial cells treated with fucoidans F and Fo was preserved (Figure 6). A significant decrease in size was observed in the Gram-positive bacteria, accompanied by some increase in the surface roughness that could indicate changes in the cell wall properties due to effect of fucoidans on bacteria metabolism, or significant quantities of fucoidan binding to cell surface. However, despite the strongest bacteriostatic effects of fucoidans on Gram-negative *E. coli*, no morphological changes were seen in these bacteria, possibly due to the presence of an outer membrane [44]. It therefore appears that the changes in cell morphology, observed in Gram-positive bacteria, represent the consequence of bacterial adaptation rather than the mechanism of growth inhibition by fucoidans.

There are two popular explanations for the possible mechanisms of the antibacterial action of polysaccharides, given by Yamashita et al. in 2001 [45]. According to the first, sulfated polysaccharides bind to the bacterial surface, thereby causing damage and nutrients leak. This version is confirmed by the detection of nucleic acids [40] and protein [30] released after the treatment of microorganisms with sulfated polysaccharides. An alternative hypothesis explains the antibacterial effect of fucoidans by trapping nutrients, for example, cationic minerals, in the nutrient medium by negatively charged molecules of sulfated polysaccharides, resulting in a decrease in the bioavailability of nutrients for microorganisms. Since, in our work, we did not observe a violation of the integrity of bacterial walls under the influence of fucoidans, it appears likely that the bacteriostatic effect on the bacteria studied is caused by fucoidans preventing the bacteria from receiving sufficient nutrition that leads to inhibition of their growth. This mechanism appears to be independent of the bacterial envelope organization and could provide a potential advantage for antibacterial applications of these compounds against pathogenic Gram-negative bacteria.

## 5. Conclusions

Antibacterial properties of two fucoidan preparations from the brown algae *F. vesiculosus* of the Barents Sea were investigated. For both fucoidan preparations, a bacteriostatic effect with MIC in the range between 4 and 6 mg/mL was observed on *E. coli*, *S. epidermidis*, *S. aureus,* and *B. licheniformis*, with *E. coli* being the most sensitive to each of the fucoidans. Purification of the crude fucoidan that involved treatment with CaCl_2_ resulted in a decrease in the mean molecular weight of fucoidan, changes in its monosaccharide composition, and a decrease in the sulfate and uronic acid contents, as well as a decrease in its antimicrobial activity.

## Figures and Tables

**Table 1:** The chemical composition of fucoidans F and Fo.

Fucoidan	Neutral Monosaccharides, mol%					Proteins, %	Sulfates, %	Uronic Acids, %	Fucose, %	Polyphenols, %
	Fucose	Xylose	Mannose	Glucose	Galactose					
F	59.9	11.1	4.3	13.6	11.1	0	27.6 ± 0.2	9.2 ± 0.5	28.7 ± 0.4	6.8 ± 0.4
Fo	43.1	24.3	1.7	8.9	22.0	0	18.3 ± 0.3	5.1 ± 0.6	38.6 ± 0.7	6.5 ± 0.6

**Table 2:** Molecular weight distribution of fucoidans.

Fucoidan	Peak	Mw, kDa	Area, %
F	1	2500–2600	3.0
	2	750–850	21.0
	3	200–250	50.2
	4	70–80	14.8
	5	10–15	11.0
Fo	1	1150–1250	11.5
	2	175–185	65.6
	3	10–20	22.0
	4	3.5–8	0.9

**Table 3:** The minimum inhibitory concentrations (MIC) of fucoidans F and Fo against test microorganisms, mg/mL.

Fraction	*E. coli*	*B. licheniformis*	*S. aureus*	*S. epidermidis*
F	4	6	4	4
Fo	4	6	6	6

**Table 4:** Surface roughness of bacteria before and after treatment with fucoidans.

Microorganisms	Control Ra, nm	F Ra, nm	Fo Ra, nm
*E. coli*	1.5 ± 0.3	2.1 ± 0.3	1.4 ± 0.2 ^†^
*B. licheniformis*	1.2 ± 0.1	1.6 ± 0.1 **	2.0 ± 0.2 **
*S. aureus*	1.3 ± 0.3	2.2 ± 0.2 *	2.5 ± 0.3 **
*S. epidermidis*	2.3 ± 0.4	4.1 ± 0.3 **	2.6 ± 0.5 ^†^

* Significantly different from control, *p* < 0.05 (** *p* < 0.01), ^†^ Significantly different from F treated, *p* < 0.05.

## Data Availability

The data presented in this study are available on request from the corresponding author in accordance with the State regulations and appropriate laws.

## Supplementary Materials

The following are available online at https://www.mdpi.com/2079-7737/10/1/67/s1, Figure S1: ^1^H NMR spectra of fucoidans F and Fo, Figure S2: Dynamic viscosity of fucoidans F and Fo, Figure S3: Inhibition of bacterial growth in the presence of 16 and 24 mg/mL concentrations of fucoidans F and Fo.
