# Supplementary material for: Antibacterial Properties of Fucoidans from the Brown Algae Fucus vesiculosus L. of the Barents Sea
Source: Biology (Basel). 2021 Jan 19;10(1):67. doi: 10.3390/biology10010067 (PMC7832856; doi:10.3390/biology10010067)
Supplement: Supplementary file 1 [file biology-10-00067-s001.zip › biology-1025239-suppl_/FigureS2.pdf]

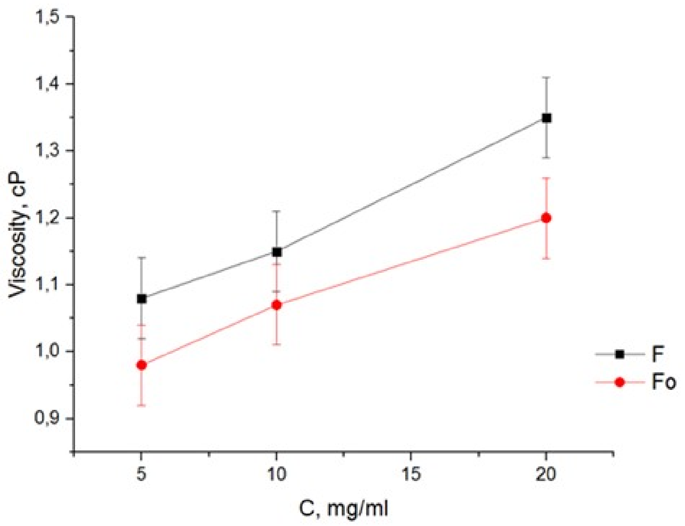

Figure S2. Dynamic viscosity of fucoidans F and Fo. Each value is the mean ± SD of three replicates ($p < 0.01$).
