# Supplementary figures and images for: Antibacterial Properties of Fucoidans from the Brown Algae Fucus vesiculosus L. of the Barents Sea
Source: Biology (Basel). 2021 Jan 19;10(1):67. doi: 10.3390/biology10010067 (PMC7832856; doi:10.3390/biology10010067)

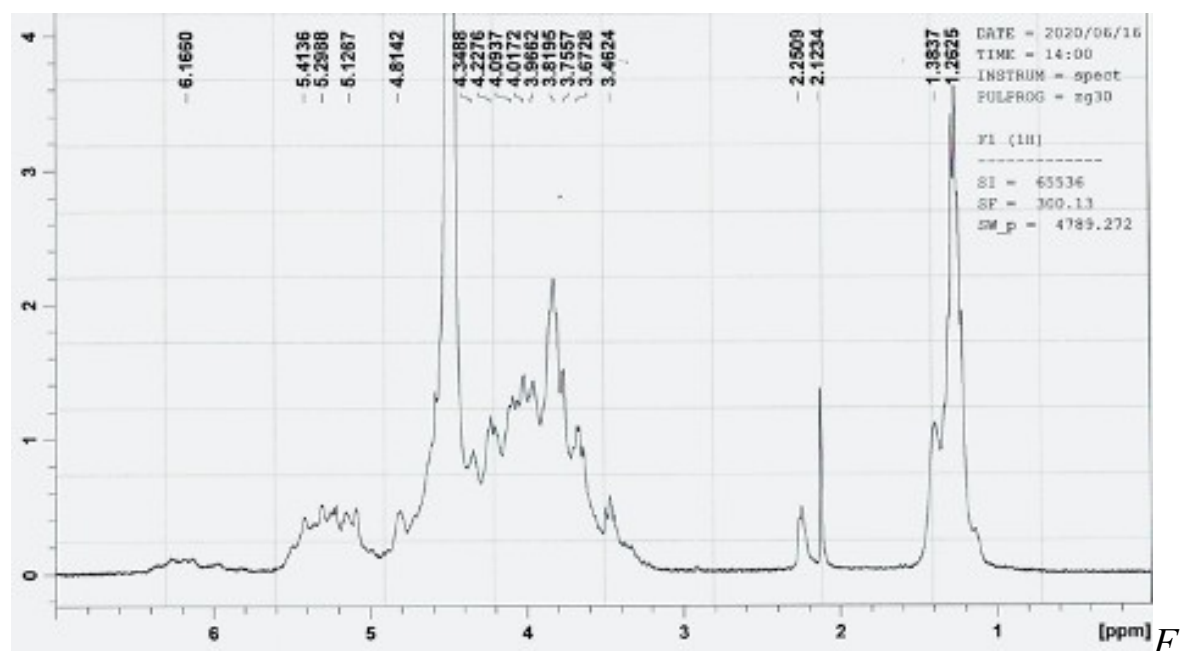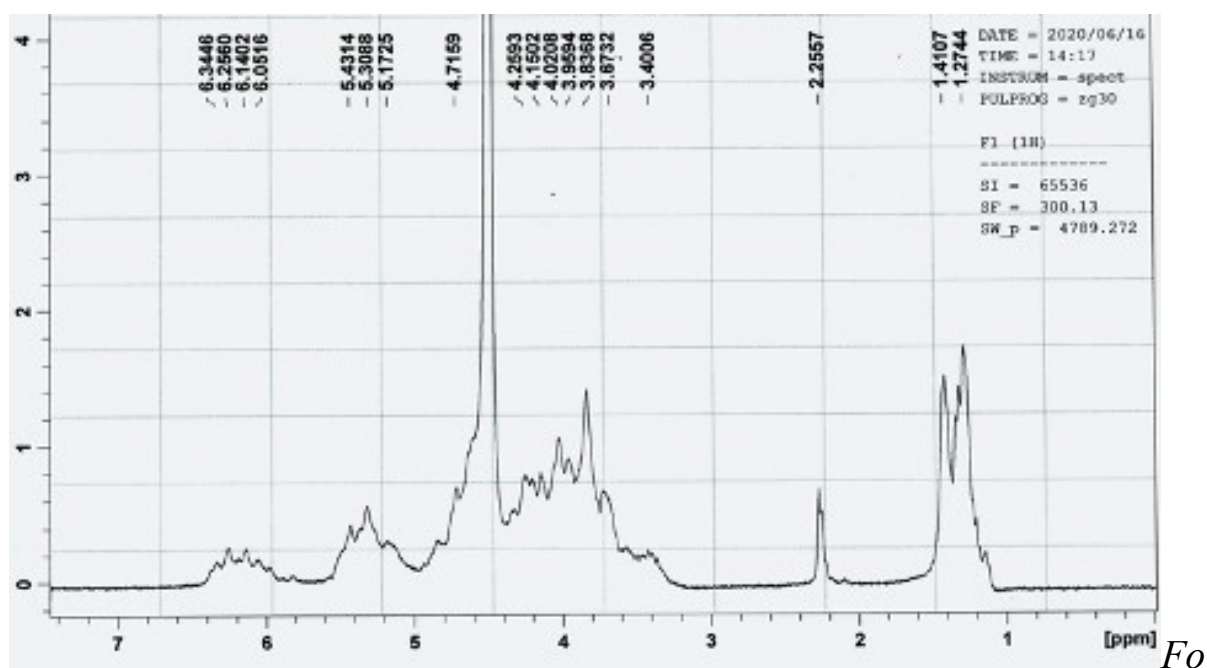

Figure S1.  $^1\text{H}$  NMR spectra of fucoidans *F* and *Fo*.

Supplement: Supplementary file 1 [file biology-10-00067-s001.zip › biology-1025239-suppl_/FigureS1.pdf]
